# Bezafibrate lowers very long-chain fatty acids in X-linked adrenoleukodystrophy fibroblasts by inhibiting fatty acid elongation

**DOI:** 10.1007/s10545-012-9471-4

**Published:** 2012-03-24

**Authors:** Marc Engelen, Martin J. A. Schackmann, Rob Ofman, Robert-Jan Sanders, Inge M. E. Dijkstra, Sander M. Houten, Stéphane Fourcade, Aurora Pujol, Bwee Tien Poll-The, Ronald J. A. Wanders, Stephan Kemp

**Affiliations:** 1 Department of Clinical Chemistry, Laboratory Genetic Metabolic Diseases, Academic Medical Center, University of Amsterdam, Amsterdam, The Netherlands; 2 Department of Neurology, Academic Medical Center, University of Amsterdam, Amsterdam, The Netherlands; 3 Department of Pediatric Neurology/ Emma Children’s Hospital, Academic Medical Center, University of Amsterdam, Amsterdam, The Netherlands; 4 Neurometabolic Diseases Laboratory, The Bellvitge Institute of Biomedical Research (IDIBELL), Center for Biomedical Research on Rare Diseases (CIBERER), Barcelona, Spain; 5 ICREA (Institució Catalana de Recerca i Estudis Avançats), Barcelona, Spain; 6 Department of Clinical Chemistry and Pediatric Neurology, Laboratory Genetic Metabolic Diseases, Academic Medical Center, Meibergdreef 9, 1105AZ Amsterdam, The Netherlands

## Abstract

X-linked adrenoleukodystrophy (X-ALD) is caused by mutations in the *ABCD1* gene encoding ALDP, an ATP-binding-cassette (ABC) transporter located in the peroxisomal membrane. ALDP deficiency results in impaired peroxisomal β-oxidation and the subsequent accumulation of very long-chain fatty acids (VLCFA; > C22:0) in plasma and tissues. VLCFA are primarily derived from endogenous synthesis by ELOVL1. Therefore inhibiting this enzyme might constitute a feasible therapeutic approach. In this paper we demonstrate that bezafibrate, a PPAR pan agonist used for the treatment of patients with hyperlipidaemia reduces VLCFA levels in X-ALD fibroblasts. Surprisingly, the VLCFA-lowering effect was independent of PPAR activation and not caused by the increase in either mitochondrial or peroxisomal fatty acid β-oxidation capacity. In fact, our results show that bezafibrate reduces VLCFA synthesis by decreasing the synthesis of C26:0 through a direct inhibition of fatty acid elongation activity. Taken together, our data indicate bezafibrate as a potential pharmacotherapeutic treatment for X-ALD. A clinical trial is currently ongoing to evaluate the effect in patients with X-ALD.

**Electronic supplementary material:**

The online version of this article (doi:10.1007/s10545-012-9471-4) contains supplementary material, which is available to authorized users.

## Introduction

X-linked adrenoleukodystrophy (X-ALD: OMIM 300100) is an inherited metabolic disorder characterized by impaired peroxisomal β-oxidation of very long-chain fatty acids (VLCFA; ≥C22) and accumulation of VLCFA (mainly ≥C26:0) in plasma and tissues of patients (Moser et al 2001). It is caused by mutations in the *ABCD1* gene (http://www.x-ald.nl), encoding a peroxisomal transmembrane protein named ALD protein (ALDP: OMIM 300371) (Mosser et al 1993). Clinically, X-ALD is characterized by a striking and unpredictable variation in phenotypic expression, ranging from the rapidly progressive childhood cerebral form (CCALD) to the more slowly progressive adult form adrenomyeloneuropathy (AMN) and variants without neurological involvement (“Addison-only” phenotype) (Moser et al 2001).

Experimental pharmacotherapy in X-ALD was aimed at normalizing VLCFA β-oxidation and VLCFA levels. Over the years several compounds have been investigated, such as Lorenzo’s oil (Aubourg et al 1993; van Geel et al 1999), 4-phenylbutyrate (Kemp et al 1998), and lovastatin (Engelen et al 2010; Singh et al 1998). These treatments were shown to be either unpractical or ineffective in clinical trials and therefore other drugs are needed.

Fenofibrate (a PPAR-α agonist) was shown to induce expression of *ALDR* (*Abcd2*) in the liver of *Abcd1* ^-/-^ mice (Netik et al 1999). ALDRP is a functional homolog of ALDP (Kemp et al 1998). However, fenofibrate has no effect on *Abcd2* expression in the brain of *Abcd1* ^-/-^ mice, possibly because it is a substrate for the *Mdr1* transporter at the blood brain barrier and therefore does not penetrate into the brain very effectively (Berger et al 1999). For this reason, we investigated the effect of several other drugs known to activate PPAR on VLCFA metabolism in cultured skin fibroblasts from patients with X-ALD. The results described in this paper show that bezafibrate (BF), but not fenofibrate, clofibrate or other PPAR agonists, could reduce VLCFA in cultured fibroblasts from patients with X-ALD.

The VLCFA which accumulate in X-ALD, are partly absorbed from the diet (Kishimoto et al 1980), but mostly result from endogenous synthesis through elongation of long-chain fatty acids (Tsuji et al 1981). Recently, we identified ELOVL1 as the key enzyme responsible for the synthesis of VLCFA and demonstrated that knock-down of ELOVL1 resulted in lower VLCFA synthesis and reduced levels of VLCFA in cultured X-ALD fibroblasts (Ofman et al 2010). Hence, inhibiting fatty acid elongation (for example by inhibition of ELOVL1) by pharmacological means could be a potential treatment for X-ALD. Here, we show that BF lowers VLCFA in X-ALD fibroblasts by direct inhibition of fatty acid elongation.

## Materials and methods

### Chemicals

Deuterium-labeled palmitate-16,16,16-D_3_ acid (D_3_C16:0) was purchased from CDN isotopes (Pointe-Claire, Canada). A 12.5 mM stock solution in dimethyl sulfoxide (DMSO) was prepared. BF, fenofibrate and clofibrate, WY14643, GW501516, and rosiglitazone were purchased from Sigma-Aldrich (St. Louis, MO, USA). Stock solutions in DMSO were made of 400 mM (BF and clofibrate), 50 mM (fenofibrate), 10 mM (WY14643 and rosiglitazone), 500 μM (GW501516). MK-886 was purchased from Cayman Chemical (Ann Arbor, MI, USA) and a stock solution of 50 mM in DMSO was used. Prior to usage the stock solutions were vortex mixed and diluted in HAMF10 tissue culture medium to the final concentration. All chemicals used were of analytical grade.

### Cell lines and cell culture

Primary human skin fibroblasts were obtained from X-ALD patients through the Neurology Outpatient Clinic of the Academic Medical Center. From each patient written informed consent was obtained. X-ALD diagnosis was confirmed by VLCFA and *ABCD1* mutation analysis. Control fibroblasts were from male anonymous volunteers. Cells from patients with a peroxisomal biogenesis disorder were obtained from the laboratory cell bank. Cells were grown in HAMF10 supplemented with 10% fetal calf serum, 2.5 mM HEPES, 100 U/ml penicillin, 100 U/ml streptomycin and 2 mM glutamine. Cells were used between passage numbers 6 and 20. Culture media were refreshed every 5 days.

### Fatty acid synthesis

Synthesis of D_3_-VLCFA in intact cells was measured using D_3_-C16:0. Assays were performed in triplicate. Cells were seeded at 40% confluency in T75 flasks. The next day, medium was replaced by fresh medium supplemented with D_3_-C16:0 (dissolved in DMSO) at a final concentration of 50 μM. After 72 hr, cells were harvested and VLCFA analyzed as described (Valianpour et al 2003).

### Measurement of fatty acid β-oxidation

Mitochondrial β-oxidation activity of intact fibroblasts was measured by quantifying the production of ^3^H_2_O from [9,10-^3^H(N)] oleic acid as described previously (Moon and Rhead 1987). Peroxisomal β-oxidation activity of intact fibroblasts was measured using [1-^14^ C]-26:0 as described previously (Wanders et al 1995). All measurements were performed in triplicate for each cell line.

### Immunofluorescence and counting of peroxisomes

Fibroblasts where grown on glass microscopy slides in 6-well plates with or without 400 μM BF. Cells were fixed with paraformaldehyde and permeabilized with Triton X-100. Peroxisomes were visualized by catalase immunofluorescence microscopy as described previously (Kemp et al 1996). To count peroxisomes, we made images of immunofluorescence-stained cells after focusing on the cell nucleus, and determined the peroxisome number per cell with the aid of a colony counter. For each cell line and condition, 10 cells were counted at random.

### Quantitative RT-PCR analysis

*ELOVL1*, *ELOVL4*, *ELOVL6*, *ACOX1*, *ABCD3* and *CPT1a* mRNA levels in control and X-ALD fibroblasts growing in log phase were determined as described (Engelen et al 2008), with primer sets presented in Table S1 (Supporting information Table S1).

### Purification of mouse liver microsomes

Microsomes were isolated from livers from wild type and transgenic ELOVL1 over-expressing mice (Kemp et al *manuscript in preparation*) by differential centrifugation as described by Baudhuin et al (Baudhuin et al 1964), with minor modifications. Livers were washed with ice-cold homogenization buffer containing 250 mM sucrose, 2 mM EDTA, 2 mM DTT and 5 mM MOPS (pH 7.4), minced and homogenized using a potter tissue grinder with Teflon pestle with 5 strokes at 500 rpm. A post-nuclear supernatant was produced by centrifugation at 600 g for 10 min. The supernatant was centrifuged at 22,500 g for 10 min and the pellet was discarded. Next, the supernatant was centrifuged for 1 h at 100,000 g to obtain a microsomal fraction. The pellet was resuspended in homogenization buffer containing 10 mg/mL methyl-β-cyclodextrin and sonicated for 4 times 5 seconds at 8 W with a 1 minute interval. The microsomal membranes were collected by centrifugation at 100,000 g for 1 h. Finally, the microsomes were resuspended in homogenization buffer and stored at -80°C until further use. All steps were carried out at 4°C. Protein concentrations were determined using BCA as described (Smith et al 1985).

### Fatty acid elongation assay

The fatty acid elongation assay was carried out using a method adapted from Nagi et al (Nagi et al 1989). The reaction mixture contained 50 mM potassium phosphate buffer (pH 6.5), 10 mg/mL α-cyclodextrin, 1 mM NADPH, 5 μM rotenone, 60 μM [2-^14^ C] malonyl-CoA (6.5 dpm/pmol) (American Radiolabeled Chemicals, St Louis, MO) and 20 μM C16:0-CoA or 20 μM C22:0-CoA (Avanti Polar Lipids, Alabaster, AL), in a total volume of 200 μL. The reaction was started by adding 100 μg protein of the microsomal fraction and allowed to proceed for 30 min at 37°C. NADPH dependency was tested by performing the reaction without NADPH. Reactions were carried out with or without BF or BF-CoA (100 – 400 μM). The reaction was stopped by adding 200 μL 5 M KOH in 10% methanol, saponified at 65°C for 1 h and acidified by adding 200 μL 5 N HCl and 200 μL 96% ethanol. Fatty acids were extracted three times with 1 mL hexane and the hexane phases were collected in a scintillation vial to which 10 mL scintillation cocktail (Ultima-Gold, Perkin Elmer) was added and radioactivity counted.

### Synthesis of bezafibroyl-CoA (BF-CoA)

BF-CoA was synthesized by a method adapted from Rasmussen et al (Rasmussen et al 1990). Dichloromethane (DCM) and tetrahydrofuran (THF) (Merck) were dried with molecular sieve deperox (Fluka). Triethylamine and ethylchloroformate (Merck) were diluted to 1 M with dry DCM. The reaction contained 36 μmol BF dissolved in 1.4 mL DCM/THF (5:2) and 40 μL 1 M triethylamine. The reaction mixture was incubated at room temperature for 10 min under constant stirring and under an atmosphere of nitrogen. After 10 min, 40 μL 1 M ethylchloroformate was added and the incubation was continued for 45 min. After the incubation, the mixture was dried under nitrogen and dissolved in 0.5 mL *tert*-butanol. Next, 40 μmol CoA trilithium salt (Sigma-Aldrich) dissolved in 0.5 mL 0.4 M potassium bicarbonate was added and the sample was incubated for 30 min at room temperature. The reaction was stopped by adding 100 μL 0.1 N HCl. BF-CoA was purified using a C18 solid phase extraction column (JTBaker). The column was eluted by a gradient of acetonitrile and 40 mM ammonium acetate, starting with 10% acetonitrile and 90% 40 mM ammonium acetate increasing to 50% acetonitrile and 50% 40 mM ammonium acetate. Acetonitrile was evaporated and purity was checked by HPLC. BF-CoA was quantified using 5,5’-dithiobis-(2-nitrobenzoic) acid (DTNB) (Sigma-Aldrich). The method used was an adaptation of that of Ellman (Ellman 1959). BF-CoA was diluted in 20 μM MES buffer pH 6.0, an equal volume of 2 M NaOH was added and the sample was incubated for 30 min at 50°C. The reaction was neutralized with 2 M HCl. Absorbance at 412 nm was measured and the concentration was determined using a calibration curve of CoA trilithium salt (Sigma-Aldrich).

## Results

### Effect of BF on endogenous VLCFA levels and *de novo* C26:0 synthesis in fibroblasts from patients with X-ALD

**Fig. 1:**
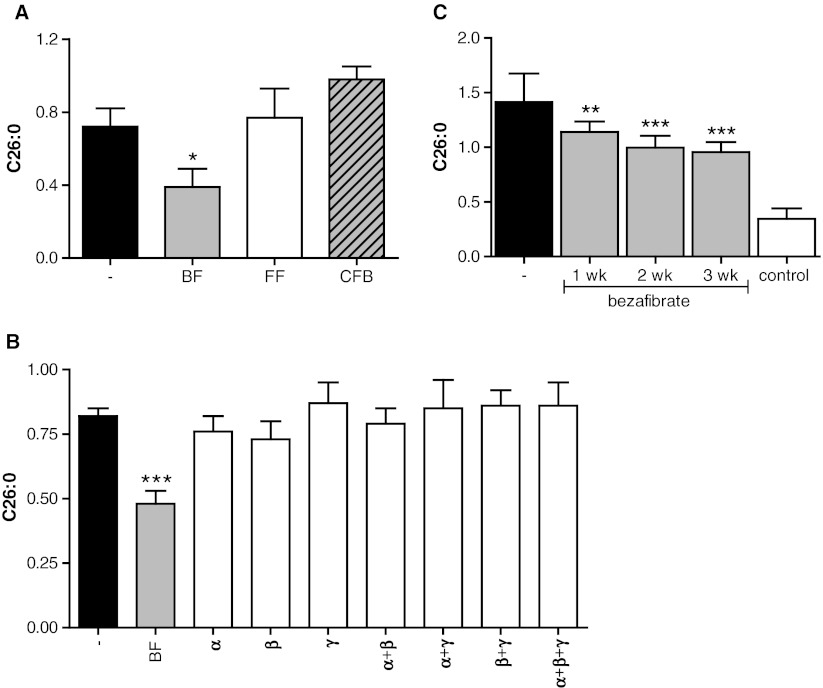
Only BF, and not other drugs from the fibrate class, reduces C26:0 in X-ALD fibroblasts. (**a**) C26:0 levels in 3 X-ALD cell lines cultured for 7 days without drugs (black bar), in the presence of BF (gray bar), fenofibrate (white bar), or clofibrate (hatched bar). (**b**) C26:0 levels in 3 X-ALD cell lines cultured for 7 days without drugs (black bar), in the presence of BF (gray bar), agonists of PPAR-α (WY 14643), PPAR-β (GW 501516) and PPAR-γ (rosiglitazon), or with different combinations of PPAR agonists (white bars). (**c**) C26:0 levels in 8 control (white bar) and 8 X-ALD cell lines cultured without (black bar) or with 400 μM BF (gray bars) for up to 3 weeks. Fatty acid levels are in nmol/mg protein. Data are mean ± SD. * = p < 0.05, ** = *P* < 0.01, *** = *P* < 0.001 by ANOVA followed by Dunnett's multiple comparison test compared with untreated X-ALD cells

We tested the effect of several drugs from the fibrate class, but found that only BF reduces C26:0 levels, both fenofibrate and clofibrate being ineffective (Fig. 1a). BF is a PPAR pan-agonist activating all three PPARs. To determine whether the effect of BF on C26:0 levels is mediated by activation of either PPARα, PPARβ/δ or PPARγ, X-ALD fibroblasts were incubated with the PPARα ligand WY14643, PPARβ/δ ligand GW501516, or the PPARγ ligand rosiglitazone either alone or in all possible combinations. Only treatment with BF reduced C26:0 levels by about 30% after 7 days (Fig. 1b).

**Fig. 2:**
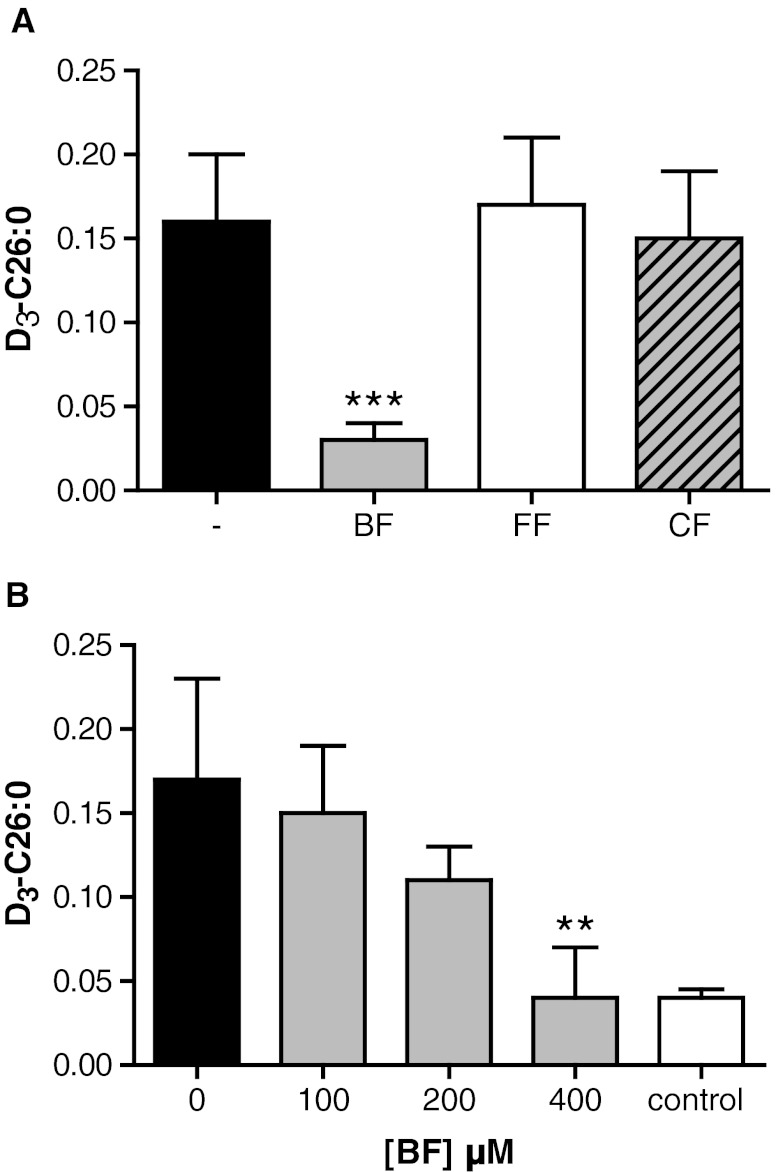
BF, but not other fibrates, inhibits D_3_-C26:0 synthesis. (**a**) D_3_-C26:0 synthesis from D_3_-C16:0 in 5 X-ALD cell lines cultured for 3 days without drugs (black bar) and in the presence of BF (gray bar), fenofibrate (FF, white bar), or clofibrate (CF, hatched bar). (**b**) D_3_-C26:0 analysis from D_3_-C16:0 in 4 control (white bar) and 4 X-ALD cell lines cultured for 3 days without (black bar) or with increasing concentrations of BF (gray bars). Fatty acid levels are in nmol/mg protein. Data are mean ± SD. ** = *P* < 0.01, *** = *P* < 0.001 by ANOVA followed by Dunnett's multiple comparison test compared with untreated X-ALD cells

To examine if longer incubations with BF would result in a further decrease of C26:0 levels, we cultured X-ALD fibroblasts for up to 21 days. This resulted in a small additional decrease of C26:0 of 10% after 14 days and 15% after 21 days, respectively (Fig. 1c). No signs of cytotoxicity or impaired growth as determined by the MTS cell proliferation assay (CellTiter 96® Aqueous One Solution Cell Proliferation Assay) were observed (data not shown). Previously, we validated the use of stable-isotope labeled fatty acids to study VLCFA *de novo* synthesis in whole cells and demonstrated that the synthesis of D_3_-C26:0 from D_3_-C16:0 is elevated in X-ALD fibroblasts (Ofman et al 2010). Earlier work has shown that BF is a potent inhibitor of long-chain fatty acid elongation, while clofibrate is not (Sanchez et al 1993; Vazquez et al 1995). Therefore, we tested the effect of BF, clofibrate and fenofibrate on C26:0 *de novo* synthesis. Of all fibrates tested, only BF affected the *de novo* D_3_-C26:0 synthesis (Fig. 2a). BF reduced D_3_-C26:0 synthesis in a concentration-dependent manner. At 400 μM BF, the synthesis of D_3_-C26:0 was reduced by 75% compared with untreated X-ALD fibroblasts (Fig. 2b). At this concentration, there was no difference in the amount of newly synthesized D_3_-C26:0 between X-ALD and control cells. We performed further experiments with BF, and not the other fibrates or PPAR agonists, to identify by which mechanism BF reduces C26:0 levels in fibroblasts from patients with X-ALD.

### The effect of BF on C26:0 levels is independent of induction of mitochondrial or peroxisomal β-oxidation

**Fig. 3:**
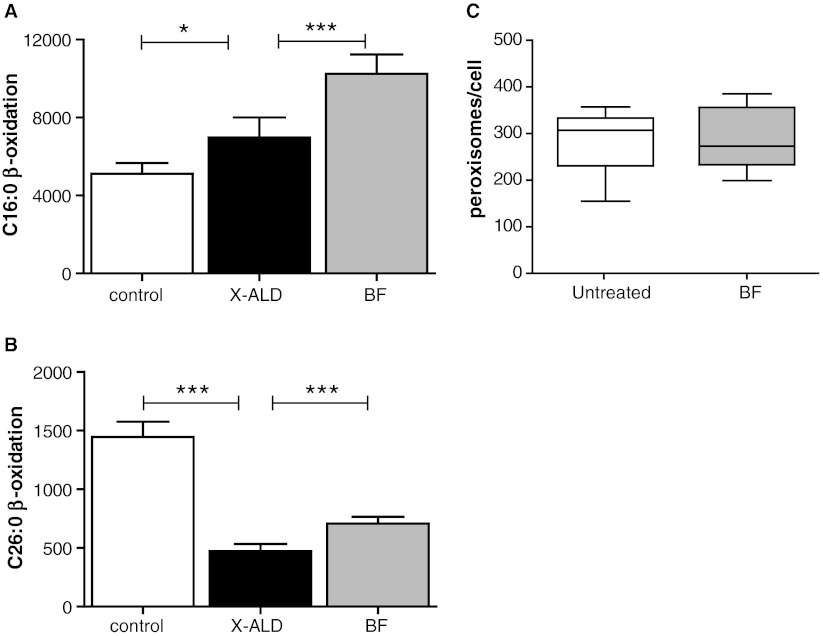
Effect of BF on mitochondrial and peroxisomal β-oxidation and peroxisome number. Measurement of (**a**) C16:0 β-oxidation and (**b**) C26:0 β-oxidation activity in 5 control cell lines (white bar) and 5 X-ALD cell lines cultured without (black bar) or with 400 μM BF (gray bar) for 7 days. Activities are in pmol/mg/hour. Data are mean ± SD. * = *P* < 0.05 and *** = *P* < 0.001 by ANOVA followed by Dunnett's multiple comparison test compared with untreated X-ALD cells. (**c**) Peroxisome number in 10 X-ALD cell lines cultured without (white bar) or with 400 μM BF (gray bar) for 3 days. Mean and quartiles are indicated, the error bars represent the range

BF and PPAR ligands in general are known to induce mitochondrial and peroxisomal β-oxidation (Bonnefont et al 2009; Cabrero et al 2001; Islinger et al 2007; Pyper et al 2010). The effect of BF treatment on the rate of mitochondrial and peroxisomal fatty acid β-oxidation was determined in X-ALD fibroblasts. Exposure of X-ALD fibroblasts to BF caused a 50% increase in C16:0 β-oxidation (Fig. 3a), and a 35% increase in C26:0 β-oxidation (Fig. 3b). Since it is known that fibrates induce peroxisome proliferation in rodents (Islinger et al 2007), we determined the amount of peroxisomes in X-ALD fibroblasts incubated for 10 days with BF. We did not find any evidence for an effect of BF treatment on peroxisome abundance (Fig. 3c).

**Fig. 4:**
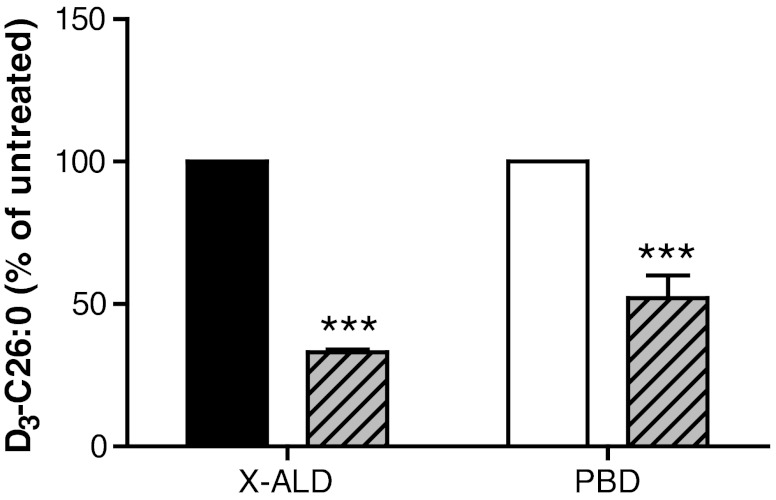
C26:0 reduction by BF is not mediated by increased peroxisomal β-oxidation. Analysis of *de novo* D_3_-C26:0 synthesis in 3 X-ALD (black bar) cell lines and 3 cell lines from patients with a peroxisomal biogenesis disorder (PBD, white bar). Cells were incubated with 50 μM D_3_-C16:0 for 3 days without or with 400 μM BF (cross-hatched bars). Fatty acid levels are in nmol/mg protein. Data are mean ± SD. *** = *p* < 0.001 by student’s unpaired *t*-test

BF reduced C26:0 levels in cultured X-ALD fibroblasts, while other drugs of the fibrate class or other synthetic PPAR agonists could not. This strongly suggested that the effect of BF on VLCFA levels is not dependent solely on induction of mitochondrial or peroxisomal β-oxidation, since the other compounds should have been effective as well then (Blaauboer et al 1990; Kemp et al 2011). BF treatment resulted in a 35% increase in the peroxisomal C26:0 β-oxidation capacity (Fig. 3b). To test if the induction of peroxisomal β-oxidation by BF caused the reduction in C26:0 levels, we incubated fibroblasts from patients with a peroxisomal biogenesis disorder (PEX1, PEX6 and PEX26) with BF and measured the effect on D_3_-C26:0 *de novo* synthesis. The synthesis of D_3_-C26:0 from D_3_-C16:0 was reduced roughly 50% in peroxisome-deficient fibroblasts incubated with BF (Fig. 4). This clearly indicated that the effect of BF on D_3_-C26:0 synthesis and C26:0 levels is only partially mediated by an induction of the peroxisomal β-oxidation capacity.

**Fig. 5:**
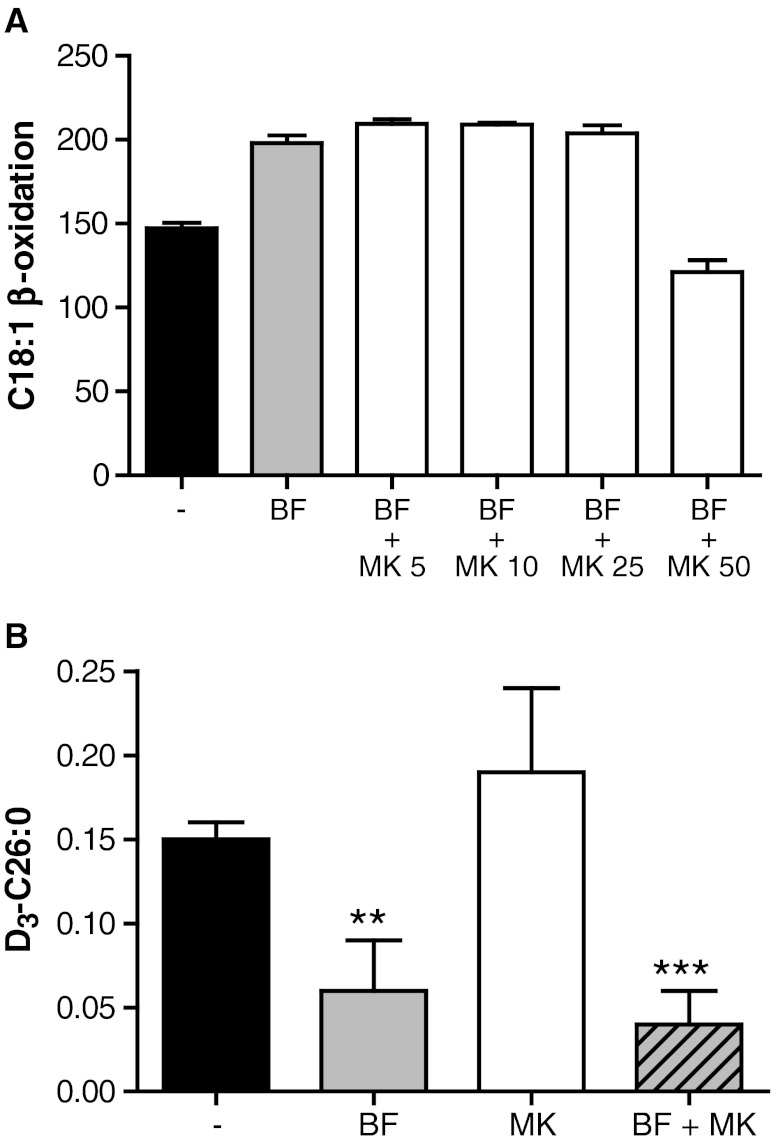
C26:0 reduction by BF is not mediated by increased mitochondrial β-oxidation. (**a**) C18:1 β-oxidation in 3 X-ALD cell lines cultured without (black bar), or for 48 hours with 400 μM BF (gray bar), or with BF and increasing concentrations of MK-886 (white bars). Activities are in pmol/mg/hour. Data are mean ± SD. (**b**) *De novo* D_3_-C26:0 synthesis in 4 X-ALD cell lines incubated with 50 μM D_3_-C16:0 without (black bar) or with 400 μM BF (gray bar), 50 μM MK-886 (white bar), or 400 μM BF and 50 μM MK-886 (cross-hatched bar). Fatty acid levels are in nmol/mg protein. Data are mean ± SD. ** = *p* < 0.01; *** = *p* < 0.001 by ANOVA followed by Dunnett's multiple comparison test compared with untreated X-ALD cells

To investigate whether the induction of mitochondrial β-oxidation by BF is responsible for the reduction in C26:0, we used the PPARα inhibitor MK-886 (Kehrer et al 2001). The induction of mitochondrial β-oxidation by BF in X-ALD fibroblasts could be reversed with 50 μM MK-886, suggesting that PPARα activation is completely blocked at this concentration (Fig. 5a). Next, we measured D_3_-C26:0 *de novo* synthesis in X-ALD fibroblasts incubated with BF in the presence of MK-886. Addition of MK-886 did not affect the reduction of D_3_-C26:0 synthesis (Fig. 5b). Combined, these data are highly suggestive that the reduction of C26:0 levels by BF is PPAR independent and can not be explained by the increase in mitochondrial β-oxidation capacity and only partially by the increase in peroxisomal β-oxidation capacity. This suggests that BF inhibits the formation of D_3_-C26:0.

### BF directly inhibits fatty acid elongation

**Fig. 6:**
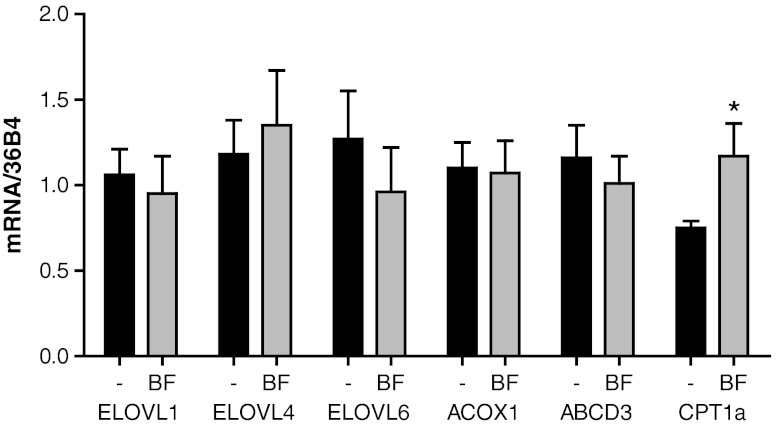
Effect of BF on gene expression of genes involved in fatty acid metabolism. Expression levels of genes involved in fatty acid metabolism in 3 X-ALD cell lines cultured without (black bars) or with 400 μM BF (gray bars) for 48 hours were analyzed by quantitative PCR. Data are mean ± SD. * = *p* < 0.05 by student’s unpaired *t*-test

The amount of D_3_-C26:0 present is the net result of the elongation of D_3_-C16:0 to D_3_-C26:0 by ELOVL6 and ELOVL1, respectively (Ofman et al 2010) and the degradation of D_3_-C26:0 by peroxisomal β-oxidation. The inhibitory effect of BF on the formation of D_3_-C26:0 could either be indirect by affecting gene expression or direct by inhibition of key enzymes involved in VLCFA *de novo* synthesis (Ofman et al 2010). To investigate the effect of BF on gene expression levels of several key enzymes involved in VLCFA synthesis, peroxisomal β-oxidation and mitochondrial β-oxidation we performed quantitative RT-PCR. As shown in Fig. 6, there is only a small reduction in the mRNA levels of *ELOVL6*, which is not statistically significant. There was no increase in the expression of *ACOX1*. This is in line with previously published data (Blaauboer et al 1990) and our own data (Fig. 3c) showing that there is no peroxisome proliferation, at least in cultured human cells, treated with fibrates, in contrast to rodents (Blaauboer et al 1990). BF did not induce the expression of *ABCD3*. Induction of this gene was detected in mice treated with fenofibrate, and considered to be the mechanism by which fibrates might be useful in correcting the metabolic defect in X-ALD (Berger et al 1999; Netik et al 1999). Our data show that induction of *ABCD3* did not occur in human X-ALD fibroblasts upon exposure to BF. In line with previous data (Djouadi et al 2005), BF treatment resulted in increased expression of *CPT1a*.

**Fig. 7:**
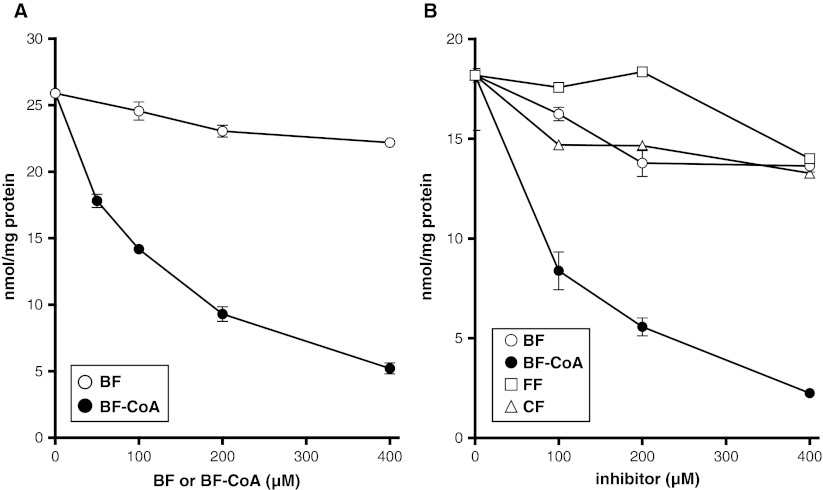
BF inhibits fatty acid elongation. (**a**) Fatty acid elongation activity of C16:0-CoA (a substrate of ELOVL6) in the presence of an increasing concentration of BF (o) and BF-CoA (●). (**b**) Fatty acid elongation activity of C22:0-CoA (a substrate of ELOVL1) in the presence of an increasing concentration of BF (o) and BF-CoA (●) or with an increasing concentration of fenofibrate (FF, □) or clofibrate (CF, ∆). Fatty acid elongation activity was measured using 20 μM C16:0-CoA or C22:0-CoA as substrate with concentrations of the inhibitor up to 400 μM. Error bars represent the standard deviation

The previous experiments suggested that the effect of BF on C26:0 levels and C26:0 synthesis could be mediated by a direct inhibiting effect on fatty acid elongation. VLCFA are synthesized by the concerted action of ELOVL6 and ELOVL1 (Ofman et al 2010). ELOVL6 elongates C16:0 to C22:0 and ELOVL1 elongates C22:0 to C26:0. We measured the effect of free BF and BF esterified to coenzyme CoA (BF-CoA) on C16:0-CoA and C22:0-CoA elongation. In the elongation assay BF had no effect. However, BF-CoA inhibited the chain elongation activity of both C16:0 and C22:0 in a concentration dependent manner (Fig. 7). Both fenofibrate and clofibrate did not inhibit C22:0-CoA elongation (Fig. 7b). These data demonstrate that BF-CoA lowers C26:0 levels by direct inhibition of fatty acid chain elongation.

## Discussion

Synthetic PPAR alpha ligands, like fibrates, are potentially interesting compounds to investigate as therapeutic agents in X-ALD because they are known to activate mitochondrial and peroxisomal fatty acid β-oxidation (Vazquez et al 2001). They might therefore reduce VLCFA accumulation by increasing VLCFA degradation. Indeed, Brown and colleagues demonstrated that treatment of two CCALD patients with clofibrate resulted in a reduction in VLCFA (Brown et al 1982). However, this reduction was not sustained. More recent experiments showed that fenofibrate induced expression of both *ALDRP* (*ABCD2*) and *PMP70* (*ABCD3*) in the liver of *Abcd1-*deficient mice, but not in brain (Berger et al 1999). In *Mdr1* ^*-/-*^ knockout mice induction of *ALDRP* and *PMP70* in brain did occur, suggesting that fenofibrate is indeed cleared from the brain by Mdr1 (Berger et al 1999). These studies, however, did not report the effect on VLCFA levels in tissues. In this paper, we studied the effect of several classical fibrates and other synthetic PPAR ligands in a cell model for X-ALD, and show that BF but not the other fibrates reduced endogenous C26:0 levels. This C26:0 reducing effect could not be mimicked by other PPAR-ligands which means that the effect is PPAR independent. BF has been demonstrated to induce mitochondrial β-oxidation (Bonnefont et al 2009; Djouadi et al 2005). Blocking the induction of mitochondrial β-oxidation with MK-886 did not prevent the reduction of D_3_-C26:0 *de novo* synthesis in X-ALD fibroblast by BF. We also showed that the peroxisomal C26:0 β-oxidation capacity in X-ALD skin fibroblasts increased with 35% upon treatment with BF. However this does not seem to be the only mechanism of reduction of VLCFA in fibroblasts incubated with BF, because in cells from patients with a peroxisome biogenesis disorder in which peroxisomal β-oxidation is completely deficient, BF lowered D_3_-C26:0 levels as well. This strongly suggested that BF might reduce C26:0 levels primarily by inhibiting C26:0 synthesis. To test this we measured *de novo* synthesis of D_3_-C26:0 from D_3_-C16:0 in X-ALD fibroblasts. BF indeed decreased *de novo* synthesis of D_3_-C26:0 in a concentration-dependent manner. At a concentration of 400 μM BF, D_3_-C26:0 levels in X-ALD fibroblasts were at the level of control fibroblasts. The rate limiting enzymes involved in synthesis of C26:0 from C16:0 are ELOVL6 (elongation of C16:0 to C22:0) and ELOVL1 (elongation of C22:0 to C26:0) (Kemp and Wanders 2010; Ofman et al 2010). By qPCR we showed that expression levels of these enzymes are not affected in fibroblasts incubated with BF suggesting a direct inhibition of VLCFA synthesis. Previously using rat liver microsomes other investigators showed that BF inhibits palmitoyl-CoA (C16:0-CoA) elongation in an *in vitro* assay (Sanchez et al 1993; Vazquez et al 1995). We used purified microsomes from wild type and ELOVL1 over-expressing mice to test the effect of BF on elongation of both long-chain fatty acids (LCFA) and VLCFA. Our results demonstrate that BF-CoA, but not free BF, is a potent inhibitor of both LCFA and VLCFA elongation. It should be noted, however, that these results do not allow us to demonstrate at which level BF inhibits VLCFA synthesis. Fatty acid elongation requires four sequential reaction steps: (i) condensation between the fatty acyl-CoA and malonyl-CoA to form 3-ketoacyl-CoA; (ii) reduction using NADPH to form 3-hydroxyacyl-CoA; (iii) dehydration to trans-2-enoyl-CoA; and (iv) reduction to fully elongated fatty acyl-CoA. The initial condensation reaction is catalyzed by the enzyme referred to as “elongation of very long-chain fatty acids” (ELOVL) and is considered to be rate limiting (Cinti et al 1992). While seven elongases have been identified in mammals (designated ELOVL1-ELOVL7)(Jakobsson et al 2006), only a single enzyme has been identified yet for the subsequent reaction step (Jakobsson et al 2006). Identification of the specific enzyme(s) affected by BF is not a trivial thing and requires detailed analysis of all enzymes involved, including: 3-ketoacyl-CoA reductase (HSD17B12), 3-hydroxyacyl dehydratase (HACD3) and the trans-2,3-enoyl-CoA reductase (TECR). This will be the subject of future studies.

### Concluding remarks

The work described in the paper shows that inhibition of VLCFA synthesis by pharmacological means could be a feasible treatment option for X-ALD. BF is a good candidate for this approach. BF lowers the levels of C26:0 by a direct inhibition of the synthesis. Mouse studies to evaluate the *in vivo* effect of BF treatment on VLCFA in X-ALD mice would be interesting; however, the effect of fibrates is quite different in rodents and humans. Watanabe et al demonstrated that rats and mice are unusable as a model system (for primates) to the study the effect of BF (Watanabe et al 1989). BF has a proven safety profile for (long-term) use in humans. With a daily dose of 200 mg of BF peak plasma levels of 50 μM can be reached, with a maximum daily dose of 800 mg of BF therapeutic levels might be reached in plasma (Miller and Spence 1998). A small scale proof of principle clinical trial is currently ongoing to evaluate the effect in X-ALD patients.

## Electronic supplementary material

Below is the link to the electronic supplementary material.

ESM 1 (DOC 39 kb)
