# Supplementary material for: Bezafibrate lowers very long-chain fatty acids in X-linked adrenoleukodystrophy fibroblasts by inhibiting fatty acid elongation
Source: J Inherit Metab Dis. 2012 Mar 24;35(6):1137–45. doi: 10.1007/s10545-012-9471-4 (PMC3470694; doi:10.1007/s10545-012-9471-4)
Supplement: Supplementary file 1 — (DOC 39 kb) [file 10545_2012_9471_MOESM1_ESM.doc]

Supplemental data

**Table S1** – primers for quantitative PCR analysis

Gene Forward primer Reverse primer

ABCD3 atgacccttggaacacttcg cgccattctttgcttttctc

ACOX1 ctgaaggctttcacctcctg catgccacacaccaactttc

CPT1a agtacacacccaaggccaag gtggatgatgctgatgatgg

ELOVL1 agcacatgacagccattcag gcttctcagttggccttgac

ELOVL2 ctcgagtcagagggtggttc cccaagtggagagaatgagc

ELOVL3 gtgctgtggtggtaccagtg gtgctgtggtggtaccagtg

ELOVL4 taagtgggttgcaggaggac ccaatggtcacatggaattg

ELOVL5 cattccctcttggttggttg tgttttccaggggtgaaaag

ELOVL6 GCTCTGGTCTCTGACCCTTG CTCCTAGTTCGGGTGCTTTG

ELOVL7 GATTGGCTCCTCATGTCCTC ATACCCCAGCCAGACATCAC
